# The Treatment of Neuralgia by Alcoholic and Other Injections

**Published:** 1916-05

**Authors:** G. Wilse Robinson

**Affiliations:** 937 Rialto Building; Kansas City, Missouri


### TEXAS MEDICAL JOURNAL

Dr. F. E. Daniel, Founder. Established July, 1885

MRS. F. E. DANIEL, - Publisher and Managing Editor

Published Monthly.—Subscription, $1.00 a Year.

VOL. XXXI AUSTIN, MAY, 1916 No. 11

The publisher is not responsible for views of contributors

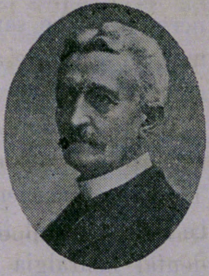

### Original Articles.

### The Treatment of Neuralgia by Alcoholic and Other Injections.

BY G. WILSE ROBINSON, M. D., KANSAS CITY, MISSOURI.

The treatment of chronic neuralgia by the internal administration of drugs is quite generally disappointing both to the patient and physician. Some twelve years ago Schlosser of Munich conceived the idea of injecting alcohol into the various branches of the trigeminal nerve at their foramina of exit for the relief of neuralgia of this nerve. The results proved to be so satisfactory that this practice has become universally popular.

In addition to Schlosser, Harris of London, Sicard of Paris, and Hertle of Berlin, have become conspicuously expert in performing this operation. Alcohol has been injected into the trunks of other nerves besides the trigeminal, but in view of the fact that it destroys motor as well as sensory nerve fibers, it should never be used in mixed or motor nerves unless used for the purpose of producing a motor paralysis.

All of the neuralgias of the trigeminal do not require alcoholic injection for their relief. The supra-orbital neuralgia resulting from malarial and other intoxications may be relieved by a dose of fifteen to twenty grains of quinine. True, dental neuralgia is a very common form of neuralgia of the trigeminal. This may be due to caries with an exposed pulp to inflamed periodontal membrane around a dead tooth and inflammation of the inferior dental nerve from direct pressure of the root of the second or third molars or to injury of the nerve in extracting these two teeth. It may be due to an infected molar or an erupting wisdom tooth. Dental neuralgia from any of these causes may be severe in type, may radiate over the entire distribution of the fifth nerve and the same side of the back of the head and neck. It may be so severe as to be mistaken for a true tic douloureux or it may cause hysterical delirium.

With this spreading neuralgia there are likely to be very definite areas of tenderness of the skin varying with the tooth affected. These areas of tenderness do not occur in tic douloureux and may be found useful in diagnostic differentiation. Local treatment of dental neuralgia must be adapted to the removal of the cause. A full dose of ten to fifteen grains of quinine sometimes will arrest at once the radiating neuralgia of dental origin or better results may be obtained by repeated doses of ten grains of butyl chloral-hydrate with five to seven grains of phenazone. This may be given hourly for three doses and then every four hours if necessary.

A supra-orbital neuralgia with pain radiating from the eyebrow up over the forehead may be due to errors of refraction or to glaucoma. Appropriate treatment will relieve. This type of neuralgia is not unusual in women during menstruation, gestation or when greatly debilitated. Quinine combined with a coal tar analgesic may give relief. Tonics should be administered but strychnine sparingly, as it frequently intensifies trigeminal neuralgia.

The more chronic type of supra-orbital neuralgia may be quickly relieved by an injection of four or five drops of 90 per cent alcohol in the supra-orbital nerve. This can be injected in the supra-orbital notch. The needle is inserted in the skin over the notch, one-fourth inch above the eyebrow. When the nerve is entered a sharp pain is felt radiating over the forehead. After injection of the alcohol a strong burning feeling is experienced spreading up over the forehead and as far back as the crown. This will be quickly followed by relief of the pain and the entire area of the distribution of the nerve will be anesthetic.

True, trigeminal neuralgia or tic douloureux is a severe and chronic form of neuralgia which affects both sexes, usually after the age of 30. Harris has observed it begin as early as the seventeenth year and as late as the eighty-first. The pain rarely affects all three branches of the nerve. The first is the least frequently affected and the second and third branches may be affected singly or together on the same side. It rarely occurs bilaterally but may do so. A thorough investigation should always be made of the teeth and sinuses, and any diseased condition found should be corrected. It has been demonstrated that a correction of dental or sinus disease has not in the majority of cases stopped the neuralgia. The reason for this is the inflammation has extended into the nerve beyond the site of the local inflammation. Removal of the local cause of irritation does not stop the inflammatory process within the nerve. The spasm of pain may be of daily occurrence, continuing for years, or it may occur more or less periodically, lasting for several weeks or months, and then disappear for a period of time. In the treatment of this condition drugs are of little value, even morphine frequently fails to give relief, and should always be used cautiously, as the danger is great that the morphine habit may be contracted. The only sure way of arresting the pain is to destroy the branch of the nerve which supplies the painful area.

The supra-orbital branch of the ophthalmic division of the trigeminal is the only one of this division affected, and can be injected in the supra-orbital notch as previously described.

The second division of the fifth nerve is injected at its exit through the foramen rotundum through the cheek just in front of the condyle of the lower jaw, using a needle of 8-9 centimeters long and 1.2 millimeters in diameter with a short point. The needle is pushed inward and upward at an angle of about 40 degrees until the external pterygoid plate is reached. The point is slowly worked forward until it slips in front of the edge of this bone, is pushed inward another five or six millimeters, when the superior maxillary nerve should be struck at a depth from the surface at from five to five and one-half centimeters. If the injection is successfully made there will be an anesthesia of the skin over the cheek, the upper lip, side of nose, upper gums and palate and as far back as the middle of the soft palate on same side.

The third division should be injected at its exit through the foramen ovale. A needle 6½ centimeters in length and .8 millimeter in diameter should be used. This is pushed through the skin at a point 3.3 centimeters in front of the external auditory meatus, and between the zygoma anl zygomatic notch of the lower jaw. This should be pushed in very slightly upward and backward to a depth of 4½ centimeters. The depth at which the nerve is struck is, of course, variable, depending upon the width of skull and character of the development of the soft parts. Symptoms indicating a successful injection of this division of the nerve are anesthesia and analgesia over the corresponding side of the lower lip and chin, tongue, lower gum and over the area of distribution of the auriculo temporal branch. There is also a slight motor palsy of the temporal, masseter and pterygoid muscles and a slight stiffness on opening the jaw, but this is not severe and passes away in a few days.

In making these injections it is best that no general anesthetic be used. A few drops of 5 per cent novocaine should be injected under the skin, and as the needle passes in a little novocaine solution in front of the needle. The point of the needle is pushed in about the locality where the nerve is supposed to be located, a careful search for the nerve should be made. When the patient complains of increased pain a few drops of novocaine solution should be injected and then a few drops of alcohol. If the novocaine solution and alcohol escape from the syringe under considerable pressure a suspicion that the solution is going within the nerve is well founded, and after a few drops have been injected the area of distribution of the nerve should be tested. If a slight anesthesia and analgesia of this area is found, more alcohol is injected and the area again tested. The only positive proof that the nerve has been entered is a quickly developing anesthesia and analgesia over its area of distribution. This does not come on quickly nor is it complete, if the alcohol is injected around the nerve trunk, although the pain may be temporarily relieved. If more than one branch of the trigeminal is affected the gasserian ganglion may be injected through the foramen ovale. After the third branch has been penetrated the alcohol is injected within its sheath, if a few drops of alcohol are injected at a time until as much as one and a half cubic centimeters have been injected, it will be found that the supra-orbital area is slightly anesthetic and the area of distribution of the middle branch decidedly so. Or it may be preferable to push the needle through the foramen ovale into the gasserian ganglion after an injection of alcohol has been made into the inferior branch. The alcohol should be injected slowly into the gasserian ganglion until the supra-orbital area is slightly anesthetic. The cells of the ophthalmic division are more deeply situated than those of the other two divisions and are the last reached by the alcohol. If sufficient alcohol is injected to cause profound anesthesia and analgesia over the supra-orbital area, ulcer of the cornea and perhaps complete destruction of the eyeball may result.

Certain accidents may result while making these injections. If a hemorrhage occurs the needle should be removed and pressure made at the point of puncture, and all blood thoroughly cleaned from the needle. After a short period of time the operation may be resumed. While endeavoring to inject the inferior branch, the pharynx may be punctured so that the injected solution will be tasted in the back of the throat. The eustachian tube may be penetrated and a sharp pain be felt in the ear. If the alcohol be injected in the subarachnoid space, pain is felt in the back of the head.

For a neuralgia radiating from the base of the nose back over the scalp into the shoulders and arms, relief may be had by injecting Meckle's ganglion through the spheno palatine foramen. In making this injection it is necessary to use a curved needle. No shock follows injection of any of the branches or of the ganglia of the trigeminal. The patient is able to be up and around within a few minutes after the work is completed. An injection within the nerve should give relief from twelve to eighteen months. After this period of time the fibers are reconstructed and the neuralgia may recur. If the alcohol is forced into the ganglion and the cells destroyed, the relief is certain to be permanent. In injecting the inferior branch of the trigeminal at the foramen ovale, I always use a considerable quantity of alcohol up to one and one-half cubic centimeters and attempt to destroy the cells of the third branch.

Brachial neuralgias are of common occurrence, are painful and disabling in character. A search is made around the scapula, painful spots are usually to be found and are tender upon deep pressure and from which the pain radiates to the shoulder and arms. The needle is pushed down to the bone and a few drops of 5 per cent novocaine solution is injected, followed by a few drops of alcohol, the neuralgic symptoms will usually rapidly disappear.

Occipital neuralgia involving the area of distribution of the great occipital nerve may be very intractable, refusing to yield to application of internal or external analgesics. The injection of the nerve gives immediate relief. The occipital is injected on a horizontal level with the external auditory meatus three-quarters of an inch from the median line. The radiating pain darting up to the crown indicates that the needle has penetrated the nerve and a few drops of novocaine solution are injected, followed by five to ten drops of 90 per cent alcohol.

Post-herpetic neuralgia. This form of neuralgia may occur in severe form in olderly people over sixty. The intensity of the pain has no relation to the extent or degree of scarring of the skin. This pain is usually resistant to internal medication and local application. The injection of alcohol down to the intervertebral foramen may arrest the pain. Failure to give relief by this method a laminectomy and section of the posterior nerve roots is the only treatment which offers hope of success.

Sciatica is a form of neuralgia that ofttimes resists internal medication. The cause of the condition is usuallly a fibrosytis of either rheumatic origin or the result of a fall, or some muscular strain. If the pain does not yield to medication an injection in the nerve may be made. The sciatic being a mixed nerve it is not advisable to use alcohol. An injection in this nerve of normal saline solution will ofttimes give immediate relief. I prefer to make the injection in the great sciatic notch. The needle is inserted three and one-half to four inches horizontally outward from the top of the intergluteal fold. The nerve is found at a depth of two and one-half to four inches. When it is penetrated a sharp stinging pain is felt radiating down the leg and into the foot. A cubic centimeter of 2 per cent novocaine is injected into the nerve, without removing the needle, a larger syringe is attached and a hundred cubic centimeters of warm sterile saline solution is injected. The effect is to inflate the nerve locally, break down adhesions and separate out the fibers. Immediately following the injection there is experienced a warm swollen sensation of the foot and leg and usually the immediate disappearance of the sciatic pain. The patient should remain in bed for twelve hours after the injection.

Painful heel. Before any form of treatment is instituted for this not uncommon type of neuralgia a careful search should be made for a bony spike growing from the under surface of the calcaneum. Foreign bodies, inflamed bursae under the tendo-achilles, etc. In the absence of any of these causes an injection of novocaine solution, followed by one cubic centimeter of salt solution at point of tenderness, will frequently give immediate and permanent relief, or if the point of the needle be pushed down to the bone and a few drops of alcohol injected, relief will be had.

The following brief clinical reports will best illustrate some of the points which I have been discussing:

No. 1, female, aged 78, had tic douloureux involving third branch of right trigeminal for a period of twelve years. Nerve injected at foramen ovale and alcohol forced into gasserian ganglion until supra-orbital area slightly anesthetic and analgesic. Complete relief of all symptoms immediately followed.

No. 2, female, aged 45, had spasmodic neuralgia of inferior branch of right trigeminal for a period of five years. This patient injected in July, 1913, but did not get complete anesthesia and analgesia over area of distribution. Pain relieved for a period of three months, after which it recurred. Another injection made in November, 1914, anesthesia and analgesia over area of distribution immediately complete. There has been no recurrence of pain and the area of the distribution of the nerve is still anaesthetic and analgesic. I am satisfied that the first injection was made around the nerve and not within the nerve sheath. In making the last injection I forced considerable alcohol within the nerve sheath and into the gasserian ganglion, that is why after a period of sixteen months there is no return of sensation over the area of distribution of this branch of the nerve.

No. 3, male, aged 34, chronic neuralgia of spasmodic type of the inferior branch of the right trigeminal covering a period of three years. Injection made at the foramen ovale. Anaesthesia and analgesia immediately followed and was complete over area of distribution of this branch. All symptoms disappeared at once.

No. 4, male, aged 45, neuralgia of spasmodic type over area of distribution of the infra-orbital branch of the trigeminal for a period of five years. The injection of the nerve within the infra-orbital foramen gave immediate relief of all symptoms with total anesthesia and analgesia over area of distribution.

No. 5, male, aged 65, had chronic neuralgia of spasmodic type involving the infra-orbital branch of the left trigeminal and the inferior branch covering a period of fifteen years. During that time he had ten injections made within the face, but after none of these injections were the lips numb. He had four injections made at Rochester, Minn., one year ago. This relieved the pain for a period of but three months. An injection was made of the infra-orbital branch in the infra-orbital foramen. Total anesthesia and analgesia immediately followed, and the upper lip felt numb to the patient. All symptoms referable to this branch relieved. On the following day an injection of the inferior branch was made at the foramen ovale. Alcohol forced within the gasserian ganglion. A complete anesthesia over the area of distribution of the inferior branch immediately followed. A needle stuck through the lower lip on the left side was not felt at all. Lip above and below on the left side were entirely numb to the patient, said he had no sensation in them at all. Relief of all symptoms immediately followed.

No. 6, female, aged 59. Had spasmodic neuralgia, right trigeminal involving all branches for a period of fifteen years. Many injections had been made around face without relief. Inferior branch injected at foramen ovale and alcohol forced into ganglion until supra-orbital area anesthetic and area of infra-orbital branch anesthetic and analgesic. After injection area of middle branch was not totally analgesic and an injection was made in the infra-orbital foramen, an injection having previously been made into the middle branch at the foramen rotundum. Complete relief was immediately had.

In making these injections it is most important that the anesthesia and analgesia come on immediately after the injection is made, that is the only real test that the nerve sheath has been penetrated and unless the nerve sheath is penetrated, and the alcohol injected within, there may be a brief relief of pain with a recurrence within two or three months. I have not seen an injection of alcohol around the nerve sheath give relief from pain for a longer period of time than three months.

I have treated some forms of brachial neuralgia very successfully by the injection of sodium cacodylate. Painful spots around the scapula are searched for, the point of the needle pushed down to the bone and the sodium cacodylate injected. A three-grain ampoule injected every day for a few days will ofttimes be followed by complete relief of the most distressing types of this form of neuralgia.

I know of no therapeutic measure in which the results are more satisfactory to patient and physician or in which so much relief can be had so quickly and so easily as is obtained by the injection treatment of neuralgia.

937 Rialto Building.

---

Dr. C. T. Grayson, President Wilson's physician and naval aide, and Miss Alice Gertrude Gordon, of Washington, will be married within the next few months.

---

The discovery of a new serum for the treatment and prevention of eruptive typhus was announced recently by Dr. Pierre Roux, director of the Paris Pasteur Institute.

This discovery is a result of researches by Dr. Nicole, director of Tunis branch of the Pasteur Institute.

